# Realistic 3D Simulators for Automotive: A Review of Main Applications and Features

**DOI:** 10.3390/s24185880

**Published:** 2024-09-10

**Authors:** Ivo Silva, Hélder Silva, Fabricio Botelho, Cristiano Pendão

**Affiliations:** 1 CMEMS—Center for Microelectromechanical Systems, University of Minho, 4800-058 Guimarães, Portugal; 2 ALGORITMI Research Center, University of Minho, 4800-058 Guimarães, Portugal; 3 Bosch Car Multimedia Portugal, S.A., 4701-970 Braga, Portugal; 4 Department of Engineering, University of Trás-os-Montes and Alto Douro, 5000-801 Vila Real, Portugal

**Keywords:** artificial intelligence, automotive, autonomous driving, AWSIM, CARLA, cooperative driving, computer graphics, open-source, sensor fusion, simulator

## Abstract

Recent advancements in vehicle technology have stimulated innovation across the automotive sector, from Advanced Driver Assistance Systems (ADAS) to autonomous driving and motorsport applications. Modern vehicles, equipped with sensors for perception, localization, navigation, and actuators for autonomous driving, generate vast amounts of data used for training and evaluating autonomous systems. Real-world testing is essential for validation but is complex, expensive, and time-intensive, requiring multiple vehicles and reference systems. To address these challenges, computer graphics-based simulators offer a compelling solution by providing high-fidelity 3D environments to simulate vehicles and road users. These simulators are crucial for developing, validating, and testing ADAS, autonomous driving systems, and cooperative driving systems, and enhancing vehicle performance and driver training in motorsport. This paper reviews computer graphics-based simulators tailored for automotive applications. It begins with an overview of their applications and analyzes their key features. Additionally, this paper compares five open-source (CARLA, AirSim, LGSVL, AWSIM, and DeepDrive) and ten commercial simulators. Our findings indicate that open-source simulators are best for the research community, offering realistic 3D environments, multiple sensor support, APIs, co-simulation, and community support. Conversely, commercial simulators, while less extensible, provide a broader set of features and solutions.

## 1. Introduction and Related Work

Nowadays, vehicles are equipped with technologies for perception, localization, actuation, and communication. These include sensors, such as Light Detection and Ranging (LiDAR), radar, cameras, and Global Navigation Satellite System (GNSS). With the progression of ADAS and autonomous driving technologies, advanced techniques such as sensor fusion are essential for integrating data collected from these sensors. The ultimate objective is to attain Level 5 autonomous driving capabilities, as defined by the Society of Automotive Engineers (SAE) International standards [1]. Achieving this level of autonomy requires sophisticated algorithms capable of perception, positioning, and decision-making based on processed information.

**Figure 1:**
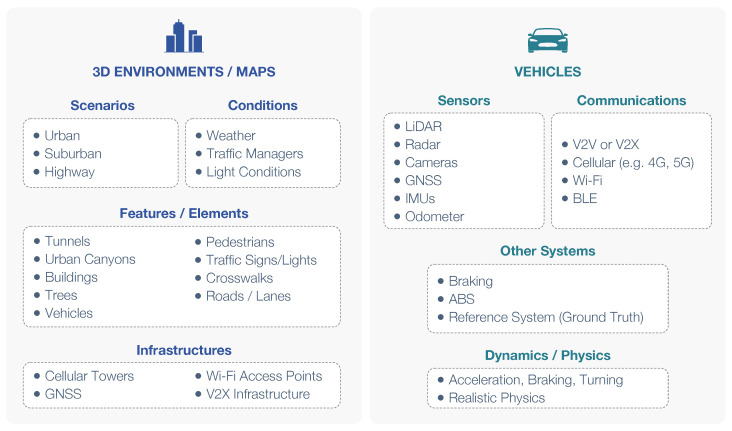
Main requirements of simulators for automotive applications.

Developing and testing autonomous driving systems or ADAS in real-world conditions poses significant challenges [2,3]. The complexity arises from the need to evaluate numerous scenarios requiring diverse environmental conditions, vehicle types, sensors, and processing techniques (Figure 1). One of the most challenging applications is the implementation of cooperative driving features, such as cooperative positioning and maneuvering [4,5]. These applications require multiple vehicles, each equipped with advanced reference systems and costly sensors, to be tested effectively in real-world settings. Another challenge is the development of solutions based on Artificial Intelligence (AI). AI techniques play a critical role in the algorithms for autonomous driving, particularly in combining multi-sensory information. However, training AI algorithms demands a substantial amount of data [6], which can be difficult and costly to acquire solely through real-world testing. Simulated data can provide an effective way for pre-training an AI model, which can then be improved with real-world data.

Simulators based on advanced computer graphics (e.g., game engines) can offer a viable solution for automotive applications by providing realistic 3D environments with visuals and vehicle physics that closely resemble the real world. They allow for testing under different conditions, including different numbers of vehicles and varying environmental factors such as weather and time of day. Moreover, these simulators support multiple sensors for perception, safety, and localization. Using these simulators allows generating large amounts of data that can be used, e.g., for prototyping new sensors, training neural networks or evaluating autonomous driving algorithms.

A survey proposed by Craighead et al. [7] analyzed computer-based simulators for unmanned vehicles, including, full-scale and micro-size vehicles, surface and subsurface vehicles, ground vehicles, and aerial vehicles. An analysis was made for commercially available and open-source simulators.

Rosique et al. [8] present a systematic review of perception systems and simulators for autonomous vehicles. The paper analyzes the necessary perception systems and sensors, details sensors for accurate positioning like GNSS and sensor fusion techniques, and reviews simulators for autonomous driving, model-based development, game engines, robotics, and those specifically designed for autonomous vehicles. A survey on simulators for self-driving cars was proposed in [9], comparing Matlab, CarSim, PreScan, Gazebo, and CARLA. The paper analyzed how well they are at simulating and testing perception, mapping and localization, path planning and vehicle control for self-driving cars. Similarly, a review of open-source simulators for autonomous driving is proposed in [10]. It categorizes simulators according to their main application, providing researchers with a taxonomy to find simulators more suitable for a particular use. Therefore, it presents these categories of simulators: traffic flow, sensor data, driving policy, vehicle dynamics, and comprehensive simulator.

This paper stands out from previous studies by specifically focusing on 3D computer graphics-based simulators for automotive applications. The main contributions of this work are: a discussion of the main applications and requirements of the simulators for automotive contexts; a comprehensive compilation of five open-source [11,12,13,14,15,16,17,18,19] and ten commercial simulators [20,21,22,23,24,25,26,27,28,29]; a detailed analysis of the features and sensors supported by these simulators.

This paper is divided as follows. Section 2 describes the main requirements and applications of 3D realistic simulators for automotive applications. Section 3 outlines the main features of these simulators. Section 4 presents selected 3D realistic simulators, followed by a detailed comparison of features and supported sensors in Section 5. Section 6 analyzes simulation technologies used by some of the most well-known companies in the automotive industry. Finally, Section 7 provides the conclusions.

## 2. Computer Graphics Simulators for Automotive: Requirements and Applications

As depicted in Figure 1, computer graphics simulators for automotive applications, particularly in autonomous driving, require two main components: realistic 3D environments (urban, suburban, highway) with varied conditions (weather, lighting, traffic), and accurate vehicle physics/motion simulation. This includes sensor models for perception, navigation, and positioning, essential for autonomous driving. Simulators should also support Vehicle-to-Everything (V2X) communication for vehicle interactions and employ traffic management systems for vehicle mobility. While computer graphics simulators excel in rendering realistic 3D environments and physics, they often lack V2X capabilities and complex traffic and mobility models. Dedicated simulators focusing on traffic, mobility, and V2X communications can complement computer graphics simulators through co-simulation.

Computer graphics-based simulators offer a plethora of features for automotive applications by providing a realistic 3D environment, which allows supporting different types of technologies and sensors. The main applications of these simulators are as follows:

- **Autonomous Driving and ADAS**: prototyping, development and evaluation of autonomous driving systems [30,31,32]. Simulated data can be used for the development of sensors, new algorithms, and sensor fusion techniques. Similarly, with autonomous driving, ADAS systems benefit from having a simulation tool that provides a multitude of scenarios in which advanced driver assistance features can be developed.
- **AI**: Simulators generate data, which is essential for developing new methods and training AI techniques for autonomous vehicles [30,33,34].
- **Cooperative Driving**: vehicles operating cooperatively, i.e., exchanging sensor data to enable cooperative positioning, perception, awareness, or cooperative maneuvering [4,35,36].
- **V2X**: communication between vehicles and other entities, which is essential for cooperative driving (maneuvering, perception, or positioning) [15,17].
- **Motorsport**: improve vehicle development (aerodynamics, chassis systems, steering systems, etc.), improve testing efficiency, and driver training [27,37,38].

## 3. Main Simulator Features

In this section, an analysis is made of the main features provided by computer graphics simulators for automotive applications. Although each simulator is unique, with differentiated functionality, they share some features that are described next.

### 3.1. Open-Source vs. Closed-Source

Commercial simulators are typically closed-source, which limits their extensibility and restricts access to the code. Also, these simulators are paid, which adds to the financial cost. In contrast, open-source simulators offer significant advantages: they are freely accessible, fostering ease of extensibility and benefiting from community support for bug fixes. Furthermore, the open nature of their source code enhances reproducibility, enabling researchers to validate and build upon each other’s findings more effectively. Henceforth, we will compare commercial and open-source simulators, with a particular focus on the open-source ones.

### 3.2. Game Engine

Simulators for automotive applications are typically built as extensions of already existing game engines. The most well-known are the Unreal Engine and Unity. These engines provide frameworks to facilitate game development, including rendering, physics and scripting. In addition, they have advanced graphics capabilities, supporting 3D development, hence they are suitable for simulating automotive scenarios, with a realistic environment for vehicles, including roads and an actor’s interaction between vehicles and other road users.

### 3.3. Supported Sensors

Most modern vehicles are equipped with several sensors, especially the ones with ADAS and autonomous driving capabilities. Various sensors gather data about the vehicle’s surroundings and internal state, for perception, localization (absolute and relative), safety and navigation. In the following, a list of commonly found sensors, supported by computer graphics simulators, is presented.

#### 3.3.1. GNSS

GNSS provides absolute positioning by using a constellation of satellites and a receiver within the vehicle to estimate its position. Computer graphics simulators have simplistic models for GNSS, usually providing the estimated positions using a simple noise model, like additive white Gaussian noise. In automotive applications, GNSS is often combined with relative positioning techniques, like dead reckoning, to improve the accuracy.

#### 3.3.2. Inertial Measurement Unit (IMU)

IMUs are self-contained systems with a tri-axis accelerometer, gyroscope, and magnetometer to measure acceleration, angular velocity, and magnetic field. IMUs usually perform on-board processing, combining raw data from all sensors into the estimation of the device’s attitude. The representation of the device’s attitude (or orientation) can be provided in Euler angles or in quaternion form.

#### 3.3.3. Encoder (Distance)

Encoders measure rotation angle or linear displacement and are often used as odometers to measure the traveled distance.

#### 3.3.4. Light Detection and Ranging

LiDARs create a 3D map of its surroundings using a laser and a receiver. It works by emitting a short laser pulse and recording the time it takes for the pulse to be reflected. This allows the conversion of time into a target distance measurement, providing a 3D representation of the surrounding environment with high-resolution point clouds, performed, for example, in 3D mapping in outdoor environments.

#### 3.3.5. Radar

Radar sensors use radio waves to measure the distance, angle, and velocity of objects. In automotive applications, radar can be used for numerous purposes, such as adaptive cruise control; collision warning and avoidance; automatic emergency brake; and blind spot detection. In autonomous driving scenarios, radar sensors are essential to reliably detect objects and people and avoid collisions.

#### 3.3.6. Ultrasound

Ultrasound sensors use high-frequency sound waves to detect objects by measuring the time it takes for the sound waves to return after hitting an object. Then, the distance to the object is calculated based on the speed of sound. Ultrasound sensors are inexpensive when compared with radar and LiDAR but are limited to short-range operation; hence, they are primarily used in short-range detection applications.

#### 3.3.7. Cameras

Cameras capture images of the environment and can be installed in several parts of the vehicles to assist in parking and to assist in autonomous navigation. They can be of different types and have different purposes, namely: RGB cameras capture color images and are used for object detection and recognition, aiding in tasks like lane keeping and traffic sign recognition; depth cameras provide 3D data about the surroundings, essential for obstacle detection and autonomous navigation; Infrared (IR) cameras enable night vision and thermal imaging, improving visibility in low-light conditions and detecting pedestrians or animals; segmentation cameras use advanced algorithms to distinguish different elements in a scene, such as vehicles, pedestrians, and road markings, facilitating autonomous driving and ADAS; optical flow cameras detect and quantify the movement of objects in a scene by analyzing changes in pixel intensity over time; event cameras, also known as Dynamic Vision Sensors (DVSs), capture changes in a scene with high temporal resolution, enabling efficient motion detection and tracking. The main drawbacks of cameras are their sensitivity to low-light environments, adverse weather conditions, and privacy concerns.

### 3.4. SIL and HIL

Software-In-the-Loop (SIL) enables the development of software components, allowing to test them in isolation from the hardware. Simulators supporting SIL usually have a simulated Electronic Control Unit (ECU), including its software components as well as simulated sensor and actuator models, as the replacement of real hardware.

Hardware-In-the-Loop (HIL) involves evaluating the interaction between software and hardware components in a simulated environment. It allows for detecting and debugging hardware–software integration issues early in the development cycle. For instance, HIL enables testing of real ECUs in a realistic simulated setting. HIL tests are reproducible and can be automated, speeding up validation and testing processes.

Both SIL and HIL facilitate the evaluation of critical corner cases within a controlled environment.

### 3.5. Co-Simulation

A simulator that supports co-simulation means it can be coupled with other simulation tools, e.g., tools that generate traffic and mobility [39,40,41], tools that support V2X communications [42,43], or autonomous driving stacks, such as Autoware [44] or Baidu Apollo [45]. This allows extending the capabilities of the computer graphics simulator to support new features that were not previously supported.

### 3.6. ROS Integration

Robotic Operating System (ROS) is a framework for developing, testing, and deploying robotic systems. It offers a standardized communications module with the publish/subscribe model. By having ROS integration, the simulator is capable of interacting with the ROS modules to implement features such as sensor fusion for positioning, SLAM, navigation, and perception.

### 3.7. Hardware Specifications

Since simulators are built on game engines, they require significant computing power due to their demands for rendering 3D graphics and simulating physics (moving objects in simulation, collision detection, gravity and other interactions). AI systems within the engine help create Non-Player Character (NPC) behaviors and other intelligent behaviors of road actors. Typically, the documentation for these tools provides detailed minimum and recommended system requirements, especially the Central Processing Unit (CPU) and Graphics Processing Unit (GPU) requirements. Regarding the CPU, a multi-core processor is usually required as it provides better multi-tasking capabilities, running multiple tasks in parallel. The CPU clock speed, usually defined in GHz, is also a requirement, and higher clock speeds improve performance. A dedicated (discrete) GPU is essential for rendering complex graphics and high-resolution textures. Some simulators include requirements for Application Programming Interfaces (APIs) like DirectX, OpenGL, or Vulkan, as these are fundamental for interacting with the GPU to create visual effects, handle complex calculations, and manage hardware resources effectively. Additionally, some simulators include RAM and disk storage requirements, as these tools have numerous assets that require both RAM and disk space to run properly.

## 4. Overview of Selected Simulators

In this section, we present the simulators selected for analysis in this paper. The primary selection criteria were simulators’ ability to provide realistic 3D environments for automotive applications using game engines, creating high-fidelity environments for autonomous driving development and testing. Both open-source and commercial options were considered to provide a holistic view of the available tools, particularly because open-source simulators are widely used both in academic and industrial research. They are free to use and offer open code, which can be extended, adapted, and supported by the community. Hence, open-source simulators are a suitable option for academic researchers and small teams with limited resources but are also used by the industry. Open-source simulators were identified through a literature review [8,9,10]. Commercial simulators were selected because they are more readily available and offer greater diversity in terms of functionality. They also have extended support, including patches and updates that enhance their reliability. However, these simulators are paid solutions and can be quite expensive, making them more accessible to industry, which typically has larger budgets, compared to academic researchers. Selected commercial simulators were found via literature review [7,8,9,10] and web search, in which we prioritized those offering more functionality according to the requirements and applications listed in Section 2.

The following subsections detail open-source simulators, highlighting their purposes, applications and main features. These are described in greater detail due to their extensibility and reusability by the research community. The last subsection introduces commercial simulators.

### 4.1. CARLA

**Figure 2:**
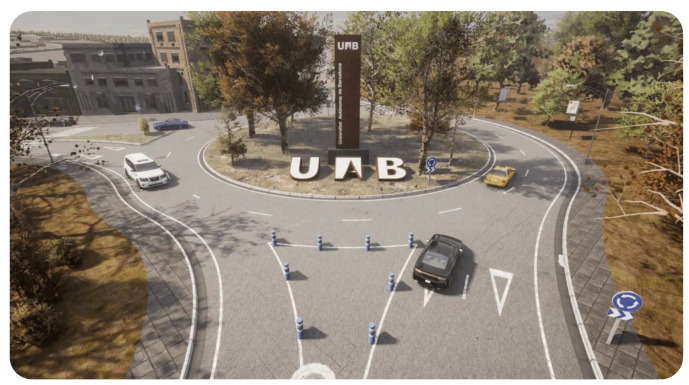
Simulated environment in CARLA simulator [11,12].

CARLA [11,12] (Figure 2) is an open-source simulator that was built specifically for autonomous driving research. It was first proposed in 2017 and is still under development with community support and feature updates. Being based on the Unreal Engine, CARLA provides a realistic 3D environment with dynamic traffic, pedestrians, and various weather conditions. The autonomous driving sensor suite provides several sensors that users can configure, e.g., LiDAR, cameras, radar, GNSS, and IMU, among others. To interact with the tool, CARLA provides an easy-to-use Python API for defining custom scenarios, controlling vehicles, and accessing sensor data.

CARLA supports co-simulation, i.e., it can be used with other simulators. It has native support for Simulation of Urban MObility (SUMO) [40], VISSIM [41], and CarSim [25]. SUMO and VISSIM are traffic and mobility simulators, which allows for managing traffic, while still being inside CARLA’s virtual environment. The integration with CarSim [25] allows vehicle controls in CARLA to be forwarded to CarSim. There are also custom co-simulation packages, developed by the research community. For instance, ref. [46] enhances CARLA with V2X capabilities, and ref. [35] improves traffic and mobility. ROS integration is enabled by a bridge that enables two-way communication between ROS and CARLA. Another important aspect of CARLA is that it has an active community on GitHub providing help not only in solving bugs and identified issues but also providing help on how to use the tool.

Being open-source and still actively supported and developed makes CARLA one of the most used open-source simulators by the research community to support cooperative perception [35], cooperative positioning [47] using LiDAR, sensor fusion applications with V2X capabilities [48], and HIL autonomous driving simulation [49].

### 4.2. AirSim

**Figure 3:**
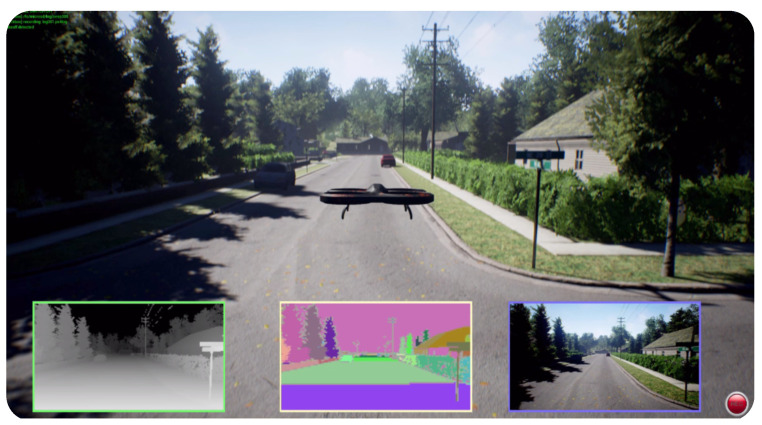
AirSim with an aerial vehicle in an urban environment [13,14].

AirSim [13,14] (Figure 3) was first introduced in 2017. It is an open-source simulator for urban environments, with realistic physics and visual rendering for drones, cars, and other vehicles. It differentiates from other simulators, since no other simulates aerial vehicles as well as land vehicles. This tool was developed by Microsoft Research using Unreal Engine as the rendering platform but also has an experimental version running with Unity. Supported sensors are a camera (RGB, infrared, optical flow), barometer, IMU, Global Positioning System (GPS), magnetometer, distance and LiDAR. Users can interact with the tool via the provided APIs in Python and C++, as well as the ROS wrapper. Another distinctive feature of AirSim is that it supports SIL and HIL, using gaming and flight controllers. In SIL mode, the algorithms development can be achieved without needing physical hardware. Conversely, in HIL mode, physical hardware can be evaluated within the simulation environment, as the simulator interfaces with the device hardware to obtain actuator signals.

Unfortunately Microsoft Research terminated this project in 2022, ending further development and support. Their decision comes from the need to focus on the development of a new product, called Project AirSim, a commercial product that provides an end-to-end platform for developing and testing aerial autonomy through simulation. Despite that, the code for AirSim is still openly available online [50].

AirSim has been used by the research community as a simulation framework, e.g., for cooperative autonomous driving using 6G V2X [51], for ADAS, with a collision avoidance system [52], and for autonomous driving based on reinforced learning [53].

### 4.3. LGSVL

**Figure 4:**
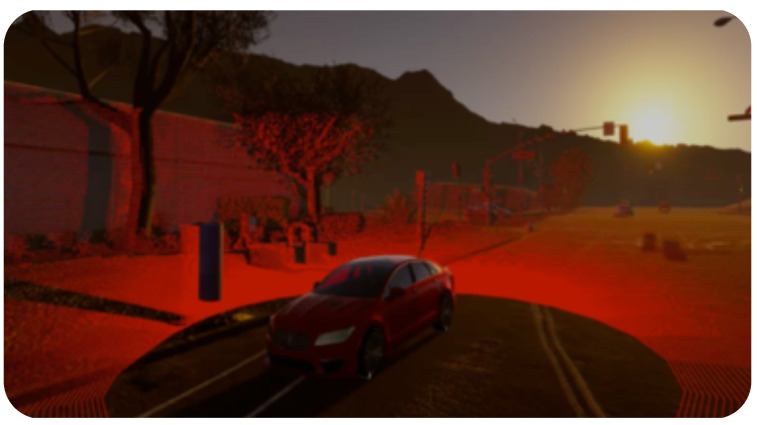
Rendering example of the LGSVL simulator [15,16].

LGSVL [15,16] (Figure 4) was developed aiming to improve autonomous vehicle development with high-fidelity simulation, exploring the Unity engine. Similarly to CARLA and AirSim, LGSVL replicates the complexity of real-world environments with the simulated environment, supporting sensors such as cameras, radar, LiDAR, GPS, IMU, among others. Supported features include SIL, HIL and ROS, as well as other integration options, particularly a communication channel that enables communication between the simulator and an autonomous driving stack, like Autoware [44] or Baidu Apollo [45]. This project was discontinued on January 1st, 2022, and no further updates or fixes are planned. Despite that, the code repository is also available online in [15].

LGSVL has been used by the research community for simulation and evaluation of autonomous driving systems. For example, in [54], a roadside-assisted cooperative planning solution developed in Autoware was evaluated using LGSVL. In [55], LGSVL was used to evaluate a camera-based perception system for autonomous driving. LGSVL was also used to evaluate a system that performs behavior monitoring of autonomous vehicles to detect safety violations [56]. This is crucial to ensuring the reliability and safety of autonomous driving systems.

### 4.4. AWSIM

**Figure 5:**
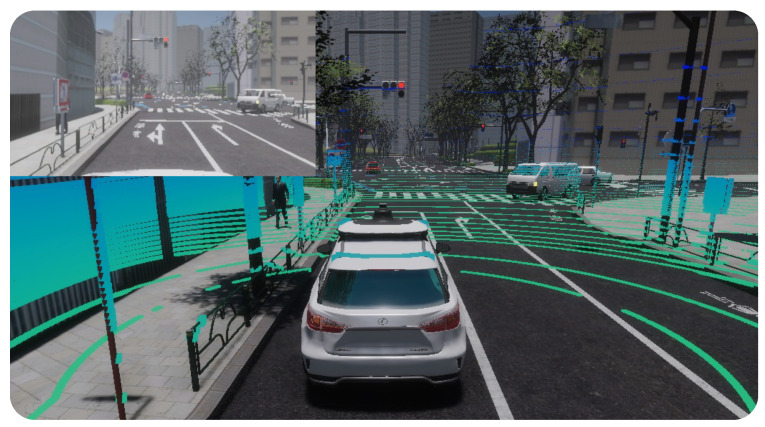
Tokyo city simulated environment in AWSIM simulator [17].

AWSIM [17] (Figure 5) was also designed for the development of self-driving capabilities in cars using the Unity engine. It supports various sensors (like LiDAR, radar, IMU, cameras, and GNSS) and accommodates different vehicle models added by users. The simulation environment offers a detailed Tokyo map as a starting point, featuring roads, buildings, traffic signals, vehicles, and pedestrians. Custom environments can also be created by users. To mimic real-world traffic, AWSIM employs a random traffic simulator that adheres to traffic rules and generates random driving paths. Built on the ROS framework, AWSIM provides sensor data, vehicle status, and other information through published topics, which allows AWSIM to be used as a scene simulator for Autoware [44].

Uses of AWSIM by the research community include supporting the development and evaluation of cooperative positioning using LiDAR-equipped roadside infrastructure for autonomous driving [57]; enabling a simulator-based automatic annotation framework that creates point cloud datasets for object detection [58] to test the latency of ROS 2 using Autoware in co-simulation, where AWSIM provides camera and LiDAR data to the Autoware autonomous driving stack [59]; creating a training dataset for a method based on deep learning to estimate localization accuracy for autonomous driving, which uses point clouds for place recognition [60].

### 4.5. DeepDrive

**Figure 6:**
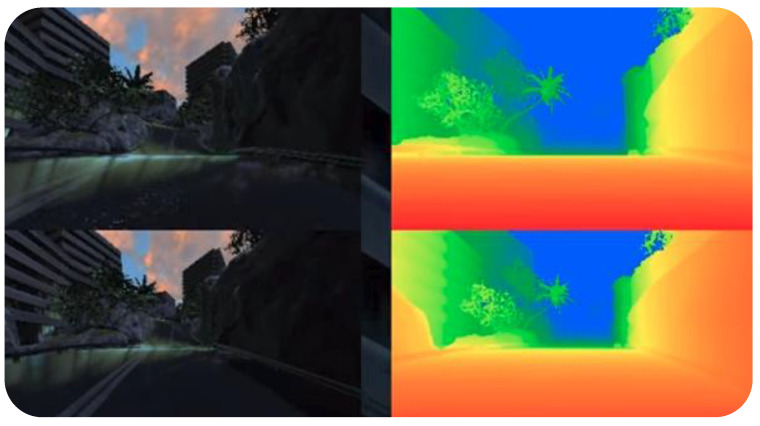
DeepDrive simulator with depth camera [18,19].

DeepDrive [18,19] (Figure 6) is another free and open-source simulator based on the Unreal Engine. It was developed mainly to support the development of AI-based self-driving capabilities using computer vision, hence it enables vehicles to be equipped with up to eight cameras with depth information. In comparison with other simulators, DeepDrive is more limited since it supports only cameras, and its documentation is not as detailed as CARLA’s or AirSim’s. DeepDrive was developed in C++, allowing the integration of existing modules using this programming language. In addition, it also provides a Python API to interact with the tool. Unfortunately, this tool was last updated four years ago.

We found no use of the DeepDrive simulator by researchers in published papers. This may indicate that the lack of features provided by this simulator makes it less appealing to the research community.

### 4.6. Commercial Simulators

**Figure 7:**
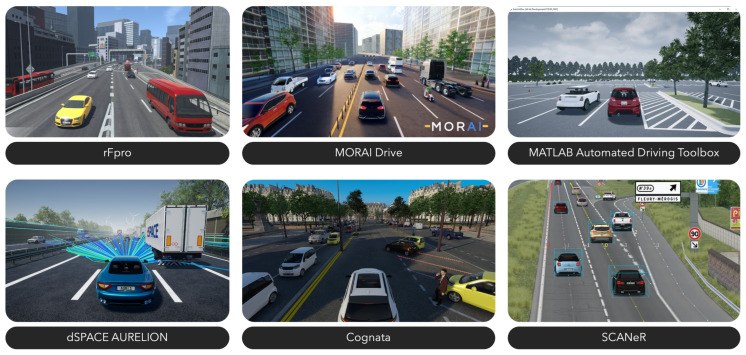
Commercial simulators [20,21,26,27,28,29].

Commercial simulators, represented in Figure 7, offer a user-friendly interface and compatibility across multiple platforms, providing a realistic environment framework for users to test their systems. Being closed-source limits extensibility and community support compared to open-source alternatives. Despite that, many commercial simulators provide open APIs to facilitate configuration and interaction. Most of these simulators are also based on game engines like Unreal Engine or Unity but integrate advanced features tailored for vehicle mobility and automated driving simulations. Ultimately, these simulators offer a comprehensive framework for automotive applications under controlled conditions.

There are many commercial simulators, including ANSYS AVxcelerate [23], Cognata [28], CarSim [25], IPG Automotive CarMaker [24], dSPACE AURELION [26], Matlab Automated Driving Toolbox [20], MORAI Drive [29], NVIDIA DRIVE Sim [22], rFpro [27], and AVSimulation SCANeR Studio [21]. Each has different characteristics and feature sets, but all focus on providing functionality for autonomous driving development, as described in Section 2.

## 5. Detailed Comparison of Simulators

In this section, an analysis and discussion about the features and sensors supported by the simulators is presented.

### 5.1. Main Features

Table 1 compares the main features of the analyzed simulators. The table includes the type of software (open/closed source), the supported operating systems, the game engine, the hardware specifications, API, SIL and HIL support, co-simulation, and ROS integration. Software can be open-source or closed-source and is identified by the main programming language used to build it. The recommended hardware specifications are provided by default. If these are unavailable, minimum specifications are provided. For graphics cards, some developers also list specific models and include requirements for API support, such as DirectX or Vulkan. This information can be found on the game engine’s website or in the simulator’s documentation.

Open-source simulators are the first on the list, followed by commercial ones. The programming language for each simulator is directly linked to the game engine it extends. For example, Unreal Engine-based simulators are developed in C++, whilst Unity-based simulators are based on C#. Some commercial simulators do not disclose their programming language, but it can be inferred from the engine used, this is the case for dSPACE AURELION, Cognata, and MORAI Drive. SCANeR, although closed-source, is considered open software because it provides a Software Development Kit (SDK) for user customization.

Apart from the cases where simulators use proprietary engines (NVIDIA DRIVE Sim, rFpro, and CarMaker), both open-source and commercial simulators are based on Unreal Engine and Unity. These engines are ideal for automotive simulators because they provide high-quality rendering, ease of use, and multiplatform support. They also have asset stores to speed up development, API integrations with other tools, and community support.

Regarding hardware specifications, most simulators require a workstation with a high-performance dedicated graphics card for rendering complex 3D environments and a multi-core CPU to support multiple processes simultaneously. RAM requirements are often tied to the demands of the graphics engine, which needs memory for large textures, models, and real-time processing. Simulations frequently involve vehicles with multiple sensors, such as cameras, generating high-resolution images at each timestep. Thus, extra RAM and disk storage are necessary to manage sensor data produced at high sampling rates.

Most simulators offer APIs for users to control specific aspects of the simulation such as the scenario, vehicles in simulation, and types of sensors, among others. Providing an API enables users to easily interact with the simulator using other existing software tools they have developed. AWSIM is the only open-source simulator that does not have API support, while the commercial simulators Matlab Automated Driving Toolbox, dSPACE AURELION, MORAI Drive, and NVIDIA DRIVE Sim, either do not provide API support or fail to mention it in their documentation. Almost all commercial simulators support SIL and HIL. CARLA and AWSIM have no native support for SIL and HIL; however, having ROS integration allows them to integrate hardware and simulation with HIL [49].

The usefulness and extendability of simulators can be evaluated by the co-simulation and ROS features. Whilst CARLA has co-simulation with traffic and mobility simulators, LGSVL and AWSIM have co-simulation with autonomous driving development tools. Half of the analyzed commercial simulators have co-simulation capabilities. Some support co-simulation with game engines like Unreal Engine, others allow integration with simulators such as CarMaker, CARLA, or dSPACE AURELION, and some offer co-simulation with traffic and mobility simulators like SUMO or VISSIM. CARLA is one of the most used simulators for co-simulation. For instance, in [61], CARLA was integrated into a co-simulation framework for Cooperative Driving Automation (CDA), in which CARLA simulates the environment, and other simulators like SUMO and Network Simulator (ns-3) simulate traffic and communications, respectively. Also, in a co-simulation framework for cooperative driving [46], CARLA was combined with Artery [43] (V2X communication) using ROS to exchange data between modules. Except DeepDrive, open-source simulators offer ROS integration, thereby extending capabilities with modules for positioning, Simultaneous Localization and Mapping (SLAM), navigation and perception. Conversely, most commercial simulators either lack ROS integration or fail to mention it in their documentation. Only the Matlab Automated Driving Toolbox and Cognata offer ROS integration.

Although the cost is not presented in Table 1, it is relevant to note that commercial simulators require a paid license, which can be considered a drawback compared to open-source simulators. For example, the Matlab Automated Driving Toolbox requires a Matlab license [62] (EUR 2250 perpetual or EUR 900 annually) plus the toolbox license [20], for which the price is unavailable. In contrast, open-source simulators are free to use, and this lack of licensing fees makes them more accessible and flexible for extension.

### 5.2. Supported Sensors

Table 2 presents a list of supported sensors by each simulator. Cameras are categorized into different types, as simulators support different types of cameras. All analyzed simulators support RGB cameras, i.e., cameras that capture colored images of the simulated environment. Many commercial simulators mention supporting cameras but do not specify which types in addition to the RGB camera. AirSim and CARLA are the open-source simulators supporting more types of cameras, showing that they are the most suited to work with computer vision, as they provide depth, segmentation, and optical flow in addition to color images. Also, AirSim supports infrared cameras, which are especially important for night-time navigation, whereas RGB cameras are not as reliable due to low visibility. Among all simulators, CARLA is the only one supporting event cameras, which provides data for improved motion detection and tracking. There are commercial simulators supporting other types of cameras, e.g., Matlab Automated Driving Toolbox supports fish-eye cameras (ultra wide angle), and Cognata supports long-distance cameras.

GNSS sensor support is provided by all open-source simulators except DeepDrive. In contrast, most commercial simulators lack GNSS sensor support, with only CarMaker, MORAI Sim Drive, and SCANeR Studio offering this functionality. It is important to note that while these simulators provide GNSS position estimates, they are based on simplistic models typically using Gaussian-distributed random noise. As such, these models do not account for satellite orbits, propagation effects and the GNSS receiver that significantly affect GNSS position estimates. Therefore, a dedicated simulator is necessary to accurately simulate raw GNSS signals (e.g., [63,64]), incorporating satellite orbits and various noise sources such as atmospheric conditions, transmitter and receiver characteristics, and multipath effects. Additionally, to simulate multipath effectively, a 3D city model and a ray tracing approach are required to achieve higher accuracy.

All open-source simulators support inertial sensors (IMUs), except DeepDrive. Conversely, most commercial simulators do not support IMUs, which are essential for tracking by providing information about the orientation. Dead reckoning algorithms and sensor fusion approaches, such as Kalman filters or Particle filters, often explore IMUs for better tracking of the vehicle’s trajectory.

Radar and LiDAR are essential for perceiving the surrounding environment. All simulators support LiDAR sensors except DeepDrive and CarSim. LiDAR 3D point clouds provide a high-resolution 3D map of the environment that enables object detection and recognition and improves navigation and path planning. Similarly, radars are used for object detection, measuring their distance, speed and direction, which allows to develop and test ADAS such as adaptive cruise control, emergency braking, and blind spot detection, among others. Most simulators support radar, except the open-source simulators AirSim, AWSIM and DeepDrive. Support for ultrasound sensors is enabled by LGSVL, Matlab Automated Driving Toolbox, CarMaker, and SCANeR Studio. Being suitable for short-range detection, ultrasound sensors are used primarily for ADAS features, such as parking assistance, blind spot detection, and collision avoidance.

Many other sensors are supported by simulators, as detailed in the last column of Table 2. These include virtual meta-sensors, such as collision, lane invasion, and obstacle sensor in CARLA; lane-line, lane following, and comfort sensor in LGSVL; traffic sign sensors and moving object detectors in CarSim; and the vision detection generator in the Matlab Automated Driving Toolbox, which detects objects using data from the vehicle’s vision sensor. These sensors enhance the development of advanced features like autonomous navigation by providing processed data rather than raw measurements. They support functionalities such as automatic lane following, adaptive cruise control, and autonomous driving using AI algorithms for traffic sign interpretation and obstacle detection. Additionally, other sensor types include magnetometers, barometers, and distance sensors in AirSim; sonar sensors in NVIDIA DRIVE Sim, and lighting sensors in SCANeR Studio.

## 6. Simulators Used by Automotive Industry

Much of the advancement in autonomous driving technology is driven by companies in the automotive industry. Many automakers, manufacturers and companies working on autonomous driving are exploring simulation to advance the development and testing of their technologies. In this section, we examine some of the most well-known companies in the automotive industry and analyze simulation technologies they are using to advance the autonomous driving industry.

The Toyota Research Institute is an investor in CARLA, having invested USD 100,000 in the further development of the tool. Bosch has used CARLA to integrate Bosch’s radar ECU software into a virtual environment [65]. Furthermore, NVIDIA has enabled Omniverse Cloud API use with CARLA, allowing users to access a platform for integrating and building custom 3D pipelines, and providing new sources of content such as vehicles, pedestrians, and many other objects.

Bosch acquired the startup company known as Five to work on simulation for developing and testing state-of-the-art software and AI-based solutions for autonomous driving [66]. Five develops state-of-the-art software and artificial intelligence-based solutions for all levels of automated driving, focusing on a platform for the development and testing of the software used in self-driving cars. This platform is capable of creating advanced testing scenarios and building a simulation environment to assess and validate system behavior at hyper-scale.

BMW partnered with ANSYS, which developed the ANSYS AVxcelerate simulator, to co-develop a simulation toolchain for autonomous/automated driving, and ADAS, supporting the generation of diverse safety scenarios and related analytics to validate system performance [67]. BMW also has teams working with Unity engine to develop graphical scenarios that simplify the testing and validation features in development [68,69]. Unity allows developers to visualize and set up thousands of simulated scenarios to validate performance under diverse conditions.

In 2022, the Stellantis Group, which owns automobile brands like Chrysler, Fiat, Jeep, Peugeot, Citroën, among others, acquired the tech startup aiMotive, aiming to accelerate autonomous driving technology [70]. aiMotive offers several solutions for autonomous driving development, including a simulation tool called aiSim. This simulator not only allows for the creation of simulated scenarios supporting multiple vehicles, sensors, and environmental conditions but also enables the creation of simulated scenarios from a real-world vehicle recording.

The PSA Group, now part of Stellantis, has used CarMaker (developed by IPG Automotive) for the development of vehicle dynamics and ADAS using SIL, HIL, and Vehicle-In-the-Loop (VIL) testing [71]. The latest development in this system involves enabling VIL, which integrates a real vehicle into a virtual traffic environment.

Hyundai is both an investor and client of MORAI Sim Drive [29], supporting its development and using it to simulate complex test scenarios based on real data.

In summary, large companies in the automotive industry typically acquire simulation tools or partner with expert companies to develop or enhance simulation tools for autonomous driving. In the future, these companies are expected to increasingly adopt existing simulation tools, extending and customizing them to meet their unique requirements. This approach speeds up the development process, allowing teams to focus primarily on the development of autonomous driving features.

## 7. Conclusions

In this paper, we reviewed several simulators based on computer graphics engines for automotive applications. These simulators are primarily used for developing, prototyping and testing ADAS and autonomous driving features, from sensor development to training AI algorithms, including cooperative driving capabilities like platooning or cooperative positioning. Computer graphics engines offer realistic 3D environments and the tools necessary to implement a simulator with support for road actors (vehicles, bicycles, pedestrians, etc.) with physics and motion dynamics.

More simulators are now available because stakeholders recognize the opportunity to provide simulation tools for the entire automotive industry. This includes car manufacturers, hardware and sensor companies, AI developers, and researchers working on automotive solutions. These simulators offer features like multiple sensor support, SIL and HIL, API, ROS integration, as well as co-simulation ability. This paper serves as a useful guide for anyone in the automotive simulation field, assisting in the selection of an appropriate simulation tool for their specific needs, whether for the development or evaluation of autonomous driving capabilities. It presents a comprehensive review of both open-source and commercial simulators, including a detailed comparison of the main features and supported sensors.

While open-source simulators offer significant advantages, such as accessibility, flexibility, and community support, they also present certain limitations. Their development often depends on small teams or community contributions, leading to delayed updates, limited support, and, in some cases, discontinuation, as seen with AirSim, LGSVL, and DeepDrive. The literature indicates numerous applications of the open-source simulators, including their use as a platform to develop and evaluate autonomous driving capabilities such as cooperative perception and/or positioning, creating datasets to train AI algorithms, and estimating localization accuracy of autonomous driving. The community benefits from having open-source simulators like CARLA because researchers can extend them to create innovative simulation frameworks that others can use [35,48].

In contrast, commercial simulators, though more robust in terms of support and updates, pose different challenges. They are typically closed-source, require paid licenses, and are less flexible or customizable. Furthermore, not all commercial simulators support key features like co-simulation, which is an important consideration when selecting a simulation tool. Many commercial options also lack transparency regarding supported features, such as APIs, co-simulation capabilities, and integration with tools like ROS. Additionally, details on supported sensors, particularly specific camera types beyond standard RGB, are often omitted.

Some simulators, like DeepDrive, are highly specialized, supporting only camera sensors. Thus, they can be used only for vision-based applications, offering limited versatility compared to other simulators designed for broader autonomous driving tasks. Consequently, their limited functionality often results in reduced interest and use from those working on autonomous driving.

Large automotive companies partner with experts on simulation technologies to advance their development in autonomous driving technologies. Typically, these companies use an existing game engine or simulation tool but develop their own simulation framework tailored to their specific validation and testing needs. As a result, these simulators are often privately owned. However, simulators like CARLA, CarMaker, aiSim, or MORAI Sim Drive are publicly available and widely used by those working in this field, benefiting from investments by private companies.

In the future, we aim to develop a cooperative positioning simulation framework. Based on this work, we select CARLA as the best open-source simulator for several reasons: (1) it has the necessary features to simulate a cooperative positioning scenario; namely, it is realistic, supports vehicles with different sensors, and allows for performing tests under different weather conditions; (2) it is still under development and being updated; (3) it has up to date and detailed documentation; (4) it has an online forum for solving issues; (5) it is free to use. We opt for an open-source simulator instead of a commercial one, firstly, because it is free to use, but also because it has community support and can be adapted and extended, thus having higher flexibility than most commercial simulators. Compared to LGSVL and AirSim, which have similar features but are discontinued, CARLA is more viable. DeepDrive, also discontinued, only supports cameras, whereas CARLA supports multiple sensor types, making it more suitable for cooperative positioning. Compared to AWSIM, CARLA offers more resources, including assets, ego-vehicles, and maps.

## Figures and Tables

**Table 1:** Comparison between computer graphics simulators for automotive applications.

Simulator	Software	OS	Engine	Specs. (GPU/CPU/RAM)	API	SIL	HIL	Co-sim.	ROS
AirSim [13]	Open source (C++, Python)	Windows, Linux, macOS	Unreal	8 GB 4-Core (2.5 GHz) 32 GB	C++, Python	✓	✓	✗	✓
AWSIM [17]	Open source (C#)	Linux	Unity	11 GB 6-Core 16 GB	—	✗	✗	✓^c^	✓
CARLA [11]	Open source (C++, Python)	Windows, Linux	Unreal	6 GB 4-Core (2.5 GHz) 32 GB	Python	✗	✗	✓^a^	✓
DeepDrive [18]	Open source (C++)	Linux	Unreal	— — 8GB	Python	—	—	—	—
LGSVL [15]	Open source (C#)	Windows, Linux	Unity	8 GB 4-Core (4 GHz) 16 GB	Python	✓	✓	✓^b^	✓
ANSYS AVxcelerate [23]	Closed source	Windows, Linux	—	— — —	C/C++, .Net, Python	✓	✓	✓^f^	—
Auto. Driving Toolbox [20]	Closed source	Windows, Linux, macOS	RoadRunner, Unreal	6 GB 4-Core (2.5 GHz) 16 GB	—	✓	✓	✓^d^	✓^e^
CarMaker [24]	Closed source (C, C++)	Windows, Linux	MovieNX, Unigine	— — —	Python	✓	✓	—	—
CarSim [25]	Closed source	Windows, Linux	—	1 GB 4-Core (2.2 GHz) 16 GB	MATLAB, VB, C/C++	✓	✓	✓^g^	✗
Cognata [28]	Closed source	MS Azure	Unity	11 GB 8-Core (3.6 GHz) 64 GB	RESTful	—	✓	✓^i^	✓
dSPACE AURELION [26]	Closed source	Windows, Linux	Unreal	12 GB 4-Core (3.8 GHz) 32 GB	—	✓	✓	—	✗
MORAI Drive [29]	Closed source	Windows, Linux, AWS	Unity	11 GB 8-Core (3.6 GHz) 64 GB	—	✗	✓	—	—
NVIDIA DRIVE Sim [22]	Closed source (C++, Python)	—	Nvidia Omniverse	— — —	—	—	—	—	—
rFpro [27]	Closed source (C++)	Windows, Linux	Proprietary	— — —	C++	✓	✓	✓^h^	✗
SCANeR [21]	Open software (C++)	Windows, Linux	Unreal	48 GB 16-Core (3.4 GHz) 64 GB	Python	✓	✓	—	—

✓: supports feature; ✗: does not support feature; —: Not applicable/not available; ^a^ SUMO [40], VISSIM [41]; ^b^ Baidu Apollo [45], Autoware [44]; ^c^ Autoware [44]; ^d^ Unreal Engine; ^e^ integration enabled by ROS Toolbox; ^f^ CarMaker [24], CARLA [11,12]; ^g^ dSPACE AURELION [26], Unreal Engine, Matlab; ^h^ SUMO [40], CarMaker [24], VISSIM [41], Matlab; ^i^ SUMO [40], Matlab.

**Table 2:** Supported sensors in vehicle simulators.

Simulator	Camera						GNSS	IMU	LiDAR	Radar	Ultras.	Others
	**RGB**	**Depth**	**Segm.**	**IR**	**OF**	**DVS**						
AirSim [13]	✓	✓	✓	✓	✓	✗	✓	✓	✓	✗	✗	✓^a^
AWSIM [17]	✓	—	—	—	—	—	✓	✓	✓	✗	✗	—
CARLA [11]	✓	✓	✓	✗	✓	✓	✓	✓	✓	✓	✗	✓^b^
DeepDrive [18]	✓	✓	✗	✗	✗	✗	✗	✗	✗	✗	✗	✗
LGSVL [15]	✓	✓	✓	✗	✗	✗	✓	✓	✓	✓	✓	✓^c^
ANSYS AVxcelerate [23]	✓	—	—	—	—	—	✗	✗	✓	✓	✗	—
Auto. Driving Toolbox [20]	✓	✓	✓	✗	✗	✗	✗	✗	✓	✓	✓	✓^d^
CarSim [25]	✓	—	—	—	—	—	✗	✗	✗	✓	✗	✓^e^
CarMaker [24]	✓	—	—	—	—	—	✓	✗	✓	✓	✓	—
Cognata [28]	✓	✗	✗	✓	✗	✗	✗	✗	✓	✓	✗	—
dSPACE AURELION [26]	✓	✓	✗	✗	✓	✗	✗	✗	✓	✓	✗	—
MORAI Sim Drive [29]	✓	—	—	—	—	—	✓	✓	✓	✓	✗	—
NVIDIA DRIVE Sim [22]	✓	—	—	—	—	—	✗	✓	✓	✓	✗	✓^f^
rFpro [27]	✓	—	—	—	—	—	✗	✗	✓	✓	✗	—
SCANeR Studio [21]	✓	—	—	—	—	—	✓	✓	✓	✓	✓	✓^g^

✓: supports sensor; ✗: does not support sensor; —: not available; **RGB**: color; **Segm.**: segmentation; **IR**: infrared (thermal camera); **OF**: optical flow; **DVS**: event camera, also known as Dynamic Vision Sensor or neuromorphic camera; **Ultras**.: ultrasound; ^a^ barometer, magnetometer, distance sensor; ^b^ collision, lane invasion, obstacle; ^c^ lane-line sensor, lane following sensor, comfort sensor; ^d^ vision detection generator (detects objects and lanes on images captured by a camera); ^e^ traffic signs sensor (camera), moving object detector; ^f^ sonar; ^g^ lighting sensor.

## Data Availability

Data sharing is not applicable.

## Abbreviations

The following abbreviations are used in this manuscript:

ADAS	Advanced Driver Assistance Systems
AI	Artificial Intelligence
API	Application Programming Interface
CDA	Cooperative Driving Automation
CPU	Central Processing Unit
DVS	Dynamic Vision Sensor
ECU	Electronic Control Unit
GNSS	Global Navigation Satellite System
GPS	Global Positioning System
GPU	Graphics Processing Unit
HIL	Hardware-In-the-Loop
IMU	Inertial Measurement Unit
IR	Infrared
LiDAR	Light Detection and Ranging
NPC	Non-Player Character
ns-3	Network Simulator
ROS	Robot Operating System
SAE	Society of Automotive Engineers
SDK	Software Development Kit
SIL	Software-In-the-Loop
SLAM	Simultaneous Localization and Mapping
SUMO	Simulation of Urban MObility
V2X	Vehicle-to-Everything
VIL	Vehicle-In-the-Loop

## References

[1] (2024). J3016_202104: Taxonomy and Definitions for Terms Related to Driving Automation Systems for On-Road Motor Vehicles-SAE International. https://www.sae.org/standards/content/j3016_202104/.

[2] Koopman P., Wagner M. (2016). Challenges in Autonomous Vehicle Testing and Validation. SAE Int. J. Transp. Saf..

[3] Lou G., Deng Y., Zheng X., Zhang M., Zhang T. (2022). Testing of autonomous driving systems: Where are we and where should we go?. Proceedings of the 30th ACM Joint European Software Engineering Conference and Symposium on the Foundations of Software Engineering.

[4] Müller F.D.P. (2015). Cooperative Relative Positioning for Vehicular Environments. Ph.D. Thesis.

[5] Aramrattana M., Larsson T., Jansson J., Nåbo A. (2019). A simulation framework for cooperative intelligent transport systems testing and evaluation. Transp. Res. Part F Traffic Psychol. Behav..

[6] Elallid B.B., Benamar N., Hafid A.S., Rachidi T., Mrani N. (2022). A Comprehensive Survey on the Application of Deep and Reinforcement Learning Approaches in Autonomous Driving. J. King Saud Univ.-Comput. Inf. Sci..

[7] Craighead J., Murphy R., Burke J., Goldiez B. A Survey of Commercial & Open Source Unmanned Vehicle Simulators. Proceedings of the IEEE International Conference on Robotics and Automation.

[8] Rosique F., Navarro P.J., Fernández C., Padilla A. (2019). A Systematic Review of Perception System and Simulators for Autonomous Vehicles Research. Sensors.

[9] Kaur P., Taghavi S., Tian Z., Shi W. A Survey on Simulators for Testing Self-Driving Cars. Proceedings of the International Conference on Connected and Autonomous Driving (MetroCAD).

[10] Li Y., Yuan W., Zhang S., Yan W., Shen Q., Wang C., Yang M. (2024). Choose Your Simulator Wisely: A Review on Open-source Simulators for Autonomous Driving. IEEE Trans. Intell. Veh..

[11] Dosovitskiy A., Ros G., Codevilla F., Lopez A., Koltun V. CARLA: An Open Urban Driving Simulator. Proceedings of the 1st Annual Conference on Robot Learning.

[12] (2024). CARLA Simulator. https://carla.org/.

[13] Shah S., Dey D., Lovett C., Kapoor A. (2017). AirSim: High-Fidelity Visual and Physical Simulation for Autonomous Vehicles. Springer Proc. Adv. Robot..

[14] (2024). AirSim Documentation. https://microsoft.github.io/AirSim/.

[15] (2024). LGSVL Simulator Github. https://github.com/lgsvl/simulator.

[16] Rong G., Shin B.H., Tabatabaee H., Lu Q., Lemke S., Možeiko M., Boise E., Uhm G., Gerow M., Mehta S. LGSVL Simulator: A High Fidelity Simulator for Autonomous Driving. Proceedings of the IEEE 23rd International Conference on Intelligent Transportation Systems ITSC.

[17] (2023). tier4/AWSIM: Open Source Simulator for Self-Driving Vehicles. https://github.com/tier4/AWSIM.

[18] (2024). DeepDrive. https://deepdrive.io.

[19] (2024). DeepDrive Github. https://github.com/deepdrive/deepdrive.

[20] (2023). Automated Driving Toolbox-MATLAB. https://www.mathworks.com/products/automated-driving.html.

[21] (2023). SCANeR Studio-AVSimulation. https://www.avsimulation.com/scaner-studio/.

[22] (2023). NVIDIA DRIVE Sim. https://developer.nvidia.com/drive/drive-sim.

[23] (2024). Ansys AVxcelerate Sensors. https://www.ansys.com/products/av-simulation/ansys-avxcelerate-sensors.

[24] (2023). CarMaker | IPG Automotive. https://ipg-automotive.com/en/products-solutions/software/carmaker/.

[25] (2024). CarSim Mechanical Simulation. https://www.carsim.com/products/carsim/.

[26] (2024). dSPACE AURELION. https://www.dspace.com/en/pub/home/products/sw/experimentandvisualization/aurelion_sensor-realistic_sim.cfm.

[27] (2023). rFpro|Simulation Software. https://rfpro.com/simulation-software/.

[28] (2024). Cognata|Autonomous and ADAS Vehicles Simulation. https://www.cognata.com/autonomous-vehicles/.

[29] (2024). Drive|MORAI Inc. https://www.morai.ai/drive.

[30] Gutiérrez-Moreno R., Barea R., López-Guillén E., Araluce J., Bergasa L.M. (2022). Reinforcement Learning-Based Autonomous Driving at Intersections in CARLA Simulator. Sensors.

[31] Osiński B., Miłoś P., Jakubowski A., Zięcina P., Martyniak M., Galias C., Breuer A., Homoceanu S., Michalewski H. (2021). CARLA Real Traffic Scenarios—novel training ground and benchmark for autonomous driving. arXiv.

[32] Gómez-Huélamo C., Del Egido J., Bergasa L.M., Barea R., López-Guillén E., Arango F., Araluce J., López J., Bergasa L.M., Ocaña M., Barea R., López-Guillén E., Revenga P. (2021). Train Here, Drive There: Simulating Real-World Use Cases with Fully-Autonomous Driving Architecture in CARLA Simulator. Proceedings of the Advances in Physical Agents II.

[33] Niranjan D., VinayKarthik B.C., Mohana Deep Learning based Object Detection Model for Autonomous Driving Research using CARLA Simulator. Proceedings of the 2021 2nd International Conference on Smart Electronics and Communication (ICOSEC).

[34] Fényes D., Hegedus T., Németh B., Gáspár P. (2021). Robust Control Design for Autonomous Vehicles Using Neural Network-Based Model-Matching Approach. Energies.

[35] Carletti C.M.R., Casetti C., Härri J., Risso F. (2024). MS-VAN3T-CARLA: An Open-Source Co-Simulation Framework for Cooperative Perception Evaluation. Proceedings of the 2024 19th Wireless On-Demand Network Systems and Services Conference (WONS).

[36] de Ponte Müller F. (2017). Survey on Ranging Sensors and Cooperative Techniques for Relative Positioning of Vehicles. Sensors.

[37] Pütz R., Serné T. (2022). Simulation of the Handling Dynamics. Race Car Handling Optimization: Magic Numbers to Better Understand a Race Car.

[38] Liu X., Fotouhi A. (2020). Formula-E race strategy development using artificial neural networks and Monte Carlo tree search. Neural Comput. Appl..

[39] Lopez P.A., Wiessner E., Behrisch M., Bieker-Walz L., Erdmann J., Flotterod Y.P., Hilbrich R., Lucken L., Rummel J., Wagner P. (2018). Microscopic Traffic Simulation using SUMO. Proceedings of the 2018 21st International Conference on Intelligent Transportation Systems.

[40] (2023). SUMO-Simulation of Urban MObility. https://www.eclipse.org/sumo/.

[41] GmbH P.P.T.V. (2024). Traffic Simulation Software|PTV Vissim. https://www.ptvgroup.com/en/products/ptv-vissim.

[42] Sommer C., German R., Dressler F. (2011). Bidirectionally coupled network and road simulation for improved IVC analysis. IEEE Trans. Mob. Comput..

[43] (2023). Artery V2X Simulation Framework. http://artery.v2x-research.eu/.

[44] (2024). Autoware Overview. https://autoware.org/autoware-overview/.

[45] (2024). Apollo. https://en.apollo.auto.

[46] Jooriah M., Datsenko D., Almeida J., Sousa A., Silva J., Ferreira J. (2024). A Co-Simulation Platform for V2X-Based Cooperative Driving Automation Systems. Proceedings of the 2024 IEEE Vehicular Networking Conference (VNC).

[47] Barbieri L., Tedeschini B.C., Brambilla M., Nicoli M. Implicit Vehicle Positioning with Cooperative Lidar Sensing. Proceedings of the ICASSP 2023—2023 IEEE International Conference on Acoustics, Speech and Signal Processing (ICASSP).

[48] Cantas M., Guvenc L. (2023). Customized Co-Simulation Environment for Autonomous Driving Algorithm Development and Evaluation. arXiv.

[49] Brogle C., Zhang C., Lim K.L., Braunl T. (2019). Hardware-in-the-Loop Autonomous Driving Simulation without Real-Time Constraints. IEEE Trans. Intell. Veh..

[50] (2024). AirSim Github. https://github.com/microsoft/airsim.

[51] Chen X., Leng S., He J., Zhou L. (2021). Deep-Learning-Based Intelligent Intervehicle Distance Control for 6G-Enabled Cooperative Autonomous Driving. IEEE Internet Things J..

[52] Liang X., Liu Y., Chen T., Liu M., Yang Q., Razavi-Far R., Wang B., Taylor M.E., Yang Q. (2023). Federated Transfer Reinforcement Learning for Autonomous Driving. Federated and Transfer Learning.

[53] Gao M., Chang D.E. Autonomous Driving Based on Modified SAC Algorithm through Imitation Learning Pretraining. Proceedings of the 2021 21st International Conference on Control, Automation and Systems (ICCAS).

[54] Hirata M., Tsukada M., Okumura K., Tamura Y., Ochiai H., Défago X. Roadside-Assisted Cooperative Planning using Future Path Sharing for Autonomous Driving. Proceedings of the 2021 IEEE 94th Vehicular Technology Conference (VTC2021-Fall).

[55] Ma C., Wang N., Chen Q.A., Shen C. (2024). SlowTrack: Increasing the Latency of Camera-Based Perception in Autonomous Driving Using Adversarial Examples. Proc. AAAI Conf. Artif. Intell..

[56] Cheng M., Zhou Y., Xie X. (2023). BehAVExplor: Behavior Diversity Guided Testing for Autonomous Driving Systems. Proceedings of the 32nd ACM SIGSOFT International Symposium on Software Testing and Analysis.

[57] Jiang Y., Javanmardi E., Tsukada M., Esaki H. (2024). Accurate Cooperative Localization Utilizing LiDAR-equipped Roadside Infrastructure for Autonomous Driving. arXiv.

[58] Zhao C., Peng B., Azumi T. Point Cloud Automatic Annotation Framework for Autonomous Driving. Proceedings of the 2024 IEEE Intelligent Vehicles Symposium (IV).

[59] Betz T., Schmeller M., Teper H., Betz J. How Fast is My Software? Latency Evaluation for a ROS 2 Autonomous Driving Software. Proceedings of the 2023 IEEE Intelligent Vehicles Symposium (IV).

[60] Matsumoto K., Javanmardi E., Nakazato J., Tsukada M. Localizability Estimation for Autonomous Driving: A Deep Learning-Based Place Recognition Approach. Proceedings of the 2023 Seventh IEEE International Conference on Robotic Computing (IRC).

[61] Xu R., Guo Y., Han X., Xia X., Xiang H., Ma J. OpenCDA:An Open Cooperative Driving Automation Framework Integrated with Co-Simulation. Proceedings of the IEEE Conference on Intelligent Transportation Systems, Proceedings, ITSC.

[62] (2024). Matlab Pricing. https://www.mathworks.com/pricing-licensing.html.

[63] (2023). Satellite Comms Toolbox. https://www.mathworks.com/help/satcom/.

[64] (2023). GPSoft|SatNav Toolbox 3.0. https://gpsoftnav.com/products/satellite-navigation-satnav-toolbox-3-0/.

[65] (2024). ADAS Simulation|Bosch Engineering. https://www.bosch-engineering.com/stories/adas-simulation/.

[66] (2024). Bosch Acquires Five. https://www.bosch-mobility.com/en/company/current-news/bosch-accelerates-software-development-for-automated-driving/.

[67] (2024). The BMW Group and Ansys Co-Developing Simulation Software for Automated and Autonomous Driving. https://www.ansys.com/news-center/press-releases/5-3-22-the-bmw-group-and-ansys-co-developing-simulation-software-for-automated-and-autonomous-driving.

[68] (2024). The Road to 240 Million Virtual Kilometers: BMW’s Autonomous Driving Journey with Unity. https://unity.com/blog/industry/road-to-240-million-virtual-kilometers-bmw-autonomous-driving-journey.

[69] (2024). Visualizing BMW’s Self-Driving Future. https://unity.com/blog/industry/bmw-automotive-lifecycle.

[70] (2024). Stellantis Accelerates Autonomous Driving Journey with Acquisition of aiMotive, a Leading Artificial Intelligence and Autonomous Driving Start-Up. https://www.stellantis.com/en/news/press-releases/2022/november/stellantis-accelerates-autonomous-driving-journey-with-acquisition-of-aimotive-a-leading-artificial-intelligence-and-autonomous-driving-start-up.

[71] (2024). Success Story Hands off, Eyes off, Mind off: New Validation Possibilities for ADAS and Autonomous Functions at PSA. https://www.ipg-automotive.com/fileadmin/user_upload/content/Download/PDF/Success_Stories/Success_Story_PSA_Vehicle-in-the-Loop_CM_EN.pdf.

